# Understanding COVID-19 Vaccine Hesitancy in Ethnic Minorities Groups in the UK

**DOI:** 10.3389/fpubh.2022.917242

**Published:** 2022-07-01

**Authors:** Maryam Naqvi, Lan Li, Michael Woodrow, Punam Yadav, Patty Kostkova

**Affiliations:** ^1^Department of Civil, Environmental and Geomatic Engineering, Univeristy College London, London, United Kingdom; ^2^Institute of Disaster Risk Reduction, University College London, London, United Kingdom

**Keywords:** vaccine, hesitancy, COVID-19, ethnic minorities, social media

## Abstract

COVID-19 vaccines have been developed and administered at record pace in order to curtail the impact of the COVID-19 pandemic. Vaccine hesitancy has impacted uptake unequally across different groups. This study explores the drivers for vaccine hesitancy in ethnic minority groups in the UK, the impact of social media on vaccine hesitancy and how vaccine hesitancy may be overcome. Twelve semi-structured interviews were conducted, coded and thematically analyzed with participants from ethnic minority groups in the UK who identified as vaccine hesitant. Social media played a significant role in vaccine hesitancy. For those who considered themselves healthy, seeing misinformation of extreme side effects relating to COVID-19 vaccinations on social media resulted in the opinion that the risk of vaccination is greater than risk from COVID-19 infection. For women, misinformation on social media regarding fertility was a reason for delaying or not getting vaccinated. Participants who had sources of information they trusted in outside of social media were more likely to choose to get vaccinated. This study identified the broad spectrum of views on vaccine hesitancy in ethnic minority groups in the UK. Enabling factors such as a desire to travel, and positive public health messaging can increase vaccine uptake, whereas a lack of trusted sources of information may cause vaccine hesitancy. Further research is required to combat misinformation and conspiracy theories. Effective methods include actively responding and disproving the misinformation. For an inclusive vaccination programme that reduces health inequality, policy makers should build trust amongst marginalized communities and address their concerns through tailored public health messaging.

## Introduction

Vaccinations are one of public health' most effective interventions, and have intersecting individual and societal benefits (1). As the COVID-19 pandemic has developed, many factors impacting vaccine uptake have come into play, such as distrust in governments (2) widespread misinformation regarding COVID-19 and concerns about the safety of vaccines due to the fast development and deployment speed (3).

Vaccine hesitancy presents a significant challenge to global public health (4) and COVID-19 has further amplified the importance of vaccine uptake. The Strategic Advisory group of Experts (SAGE) In the UK, defined vaccine hesitancy as “delay in acceptance or refusal of vaccination despite availability of vaccination services.” Vaccine hesitancy is complex and context specific, with a wide range of social and physical variables. It is influenced by factors such as complacency, convenience, and confidence, known as the “3Cs” model (5).

The success of vaccination programmes depends on where, how and what information regarding the safety, efficacy, and access of vaccinations is communicated (6). There is an association between anti-vaccination beliefs, conspiracy theories, reduced trust in institutions and an increased reliance on social media for information on health (7). A survey of 1,476 UK adults found that users of YouTube, Instagram, Snapchat, and TikTok are all less likely to express willingness for vaccination against COVID-19 (7). Examples of misinformation on social media includes links between the 5G mobile network and COVID-19, and the theory that the pandemic is a bioweapon or conspiracy (8). Recent systematic review of vaccines hesitancy and social media interventions illustrated a major gap and lack of robust evaluation results (2). As social media grows exponentially, the anti-vax movement is also expected to spread further across the multitude of platforms. The term anti-vaxxers can be defined as “someone who believes vaccines do not work, are not safe or refuse vaccines for themselves and their children if applicable” (9) and should not be used interchangeably with vaccine hesitancy.

In the UK, while surveys show over 90% of adults express positive sentiments toward the vaccine (10), when looking at ethnic minorities specifically, vaccine hesitancy increases substantially. A December 2020 survey of 12,035 participants showed the highest vaccine hesitancy in Black (71.8%), followed by Pakistani/Bangladeshi (42.4%) and Mixed (32.4%) ethnicity people. With 15.6% of White British or Irish groups showing vaccine hesitancy (11). A 2020 report from Public Health England showed that after accounting for the impact of age, region and deprivation and gender, people of ethnic minorities including Indian, Chinese, Black had between 10 and 50% higher risk of mortality from COVID-19, compared to people who were White British (12).

The study aims to build on existing research by undertaking semi-structured interviews with a convenience sample of people from ethnic minority groups who have expressed concerns regarding the Covid-19 vaccines. The following research questions were explored in this study; (1) What are the primary reasons for vaccine hesitancy in ethnic minority groups in the UK? (2) How does social media impact vaccine uptake in ethnic minority groups in the UK? (3) What enables people from ethnic minorities to overcome vaccine hesitancy? The next section, *Methodology*, details the study design, data collection and analysis. This is followed by *Results* which comprises the key findings from the coded interview transcripts. The themes identified in *Results* will then be reviewed and analyzed in *Discussion and Conclusions*.

## Methodology

### Study Design

**Figure 1:**
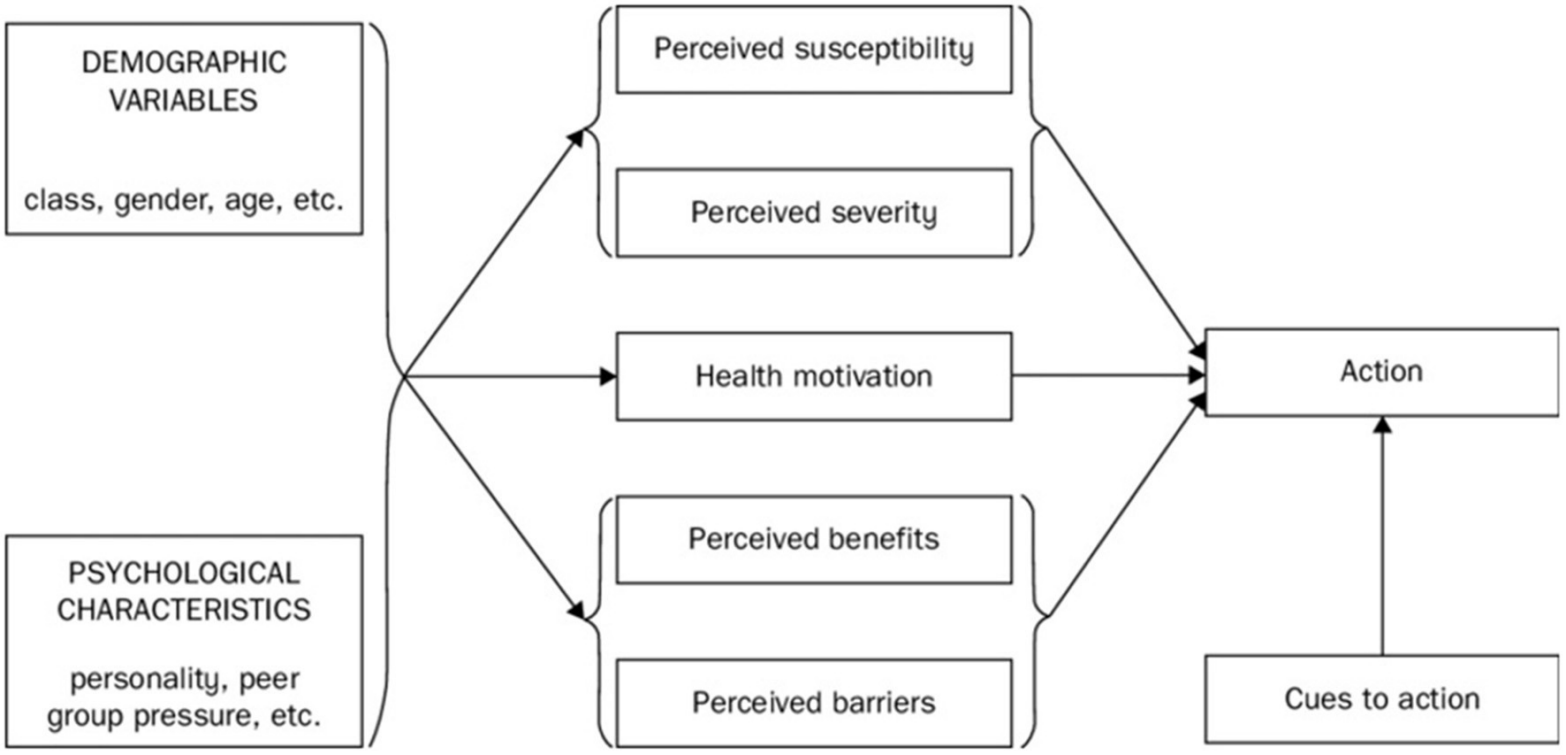
Reasons for vaccine hesitancy.

A qualitative study, comprising of twelve semi-structured interviews to identify reasons for vaccine hesitancy, the primary factors that impact vaccine hesitancy and vaccine uptake, and the role of social media in vaccine hesitancy in ethnic minority communities. Developing the interview questions was an iterative process, and began with five questions based on the Health Belief Model, which evolved into a decision tree. The Health Belief Model (HBM) is a widely used theory in understanding and predicting health behavior, and comprises of five central components, which are impacted by demographic variables and psychological characteristics, as can be seen in Figure 1 (13). Studies have demonstrated that interventions targeting the HBM constructs can improve vaccine uptake (14), hence the HBM model components have been integrated into the study interview questions. Pilot interviews which tested the relevance and usefulness of the interview questions against the research aims were also utilized. Due to COVID-19, the interviews were undertaken online on video conference platform Microsoft teams. The interviews were recorded and transcribed verbatim by the interviewer and were conducted entirely in English.

### Recruitment of Participants

Participants were invited to take part in the study based on the following inclusion criteria; over 18 years old, living in the UK, identified as part of an ethnic minority group, and have or have had concerns regarding vaccinating against COVID-19. In this study, the term “ethnic minorities” refers to all ethnic groups except the white British group. The interviews were carried out between July and August, 2021. People who identified themselves as vaccine hesitant, but have taken the COVID-19 vaccine, were included within the study to explore how and why some are able to overcome their vaccine hesitancy. The participants were recruited by snowball sampling. The interviews were confidential to encourage open and honest answers and increase participant comfort.

### Analysis Plan

Conventional content analysis (15), in which coding categories are derived directly from the text, was used to code the semi-structured interview transcripts. The Framework method was next used to organize and chart the coded, which comprised of six stages—familiarization, coding (using NVIVO 12, a Qualitative Data Analysis software), designing a thematic framework, indexing and charting. In the final stage—mapping and interpretation—the characteristics of the charted data were analyzed to review the primary themes and topics from the coded data. These themes were then used to identify gaps in the existing literature, providing theoretical triangulation and informing scope for future studies (16).

### Background of the Participants

Out of twelve participants, five had received one or both vaccinations against COVID-19 while seven participants had chosen not to be vaccinated against COVID-19. All participants resided in the UK at the time of interview. Seven participants identified as female and five identified as male. The groups represented by the participants were Black, Arab and Asian backgrounds; which included Indian, Pakistani and Asian Other.

## Results

The coding of the twelve interview transcripts was conducted in several steps. First, the interview transcripts were first coded line by line, under nodes such as “Choice,” “Infertility,” and “Skeptical” to develop an understanding of the answers. The nodes were then organized in terms of the interview questions, under categories such as “Information and Misinformation” and “Vaccine Concerns”. Following this, key themes including “Research” and “Confusion and Uncertainty” identified in the transcripts were organized and coded.

### Reasons for Vaccine Hesitancy

**Figure 2:**
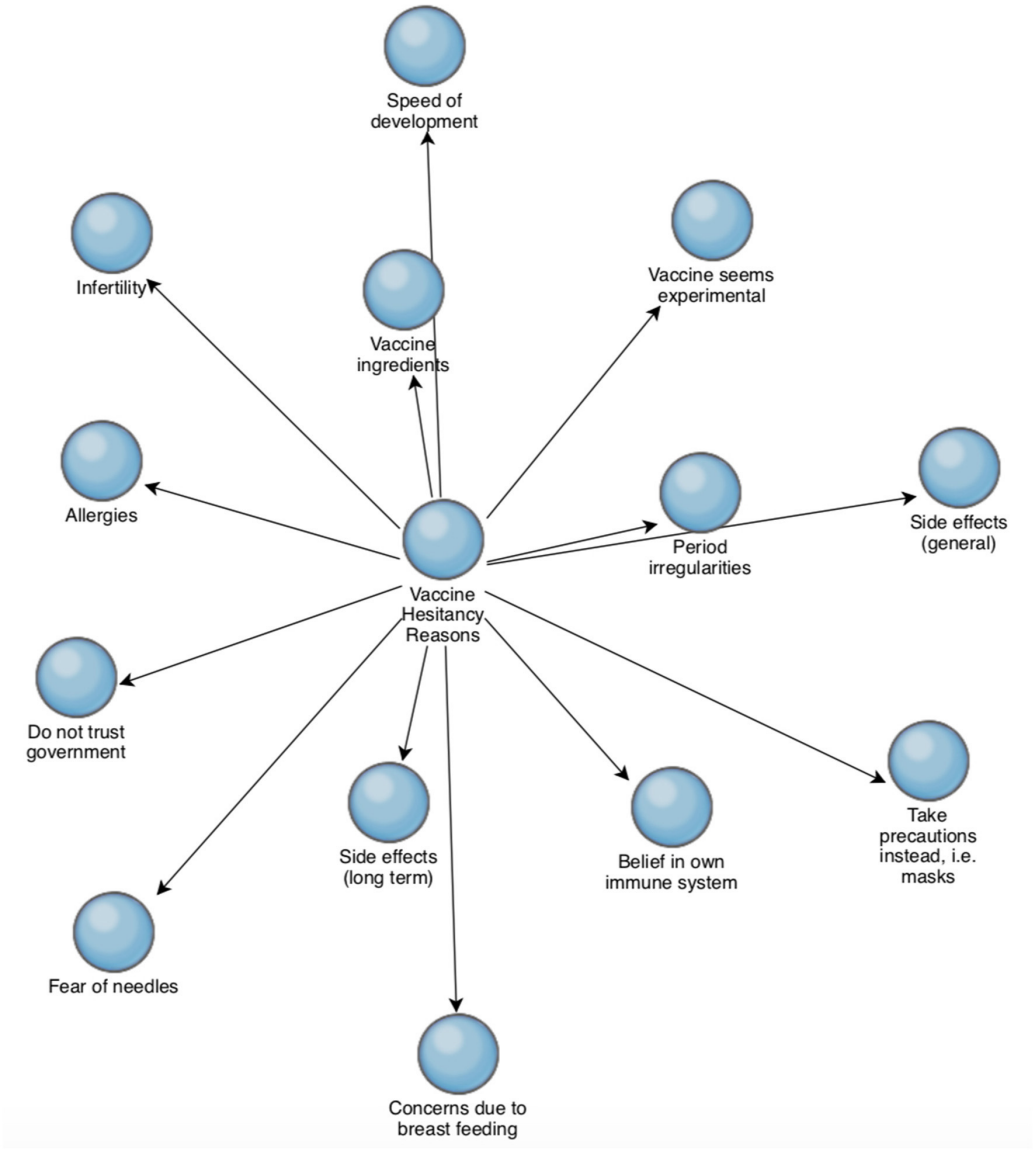
Motivations for having received the COVID-19 vaccine.

The reasons cited in the interviews for vaccine hesitancy can be seen in Figure 2. The numbers refer to the number of interviews that the reasons were cited in. General and long term side effects and speed of development of the COVID-19 vaccinations were the most common reasons, followed by the “belief in your own immune system” and concerns regarding the ingredients in the COVID-19 vaccine.

Five of the twelve participants cited both side effects and long term side effects as concerns, while two of the twelve participants cited side effects generally, and a further two participants cited long term side effects specifically as concerns.

- “*In the long term, I am thinking what is the point of putting a dormant version of the virus in me that could cause more complications down the line, because my body seems to be fighting COVID-19 fine at the moment*.” Transcript 2 (male, not vaccinated)
- “*One of my friends had a stroke after the vaccine, and they said it wasn't because of the vaccine. I feel like there is something that they are not telling us, I think it's a global thing, not specifically the UK government, I am suspicious.”* Transcript 4 (female, not vaccinated)

Concerns regarding period irregularities, infertility and breastfeeding were recurring themes in three interviews T1 (female, not vaccinated), T3 (female, not vaccinated), and T6 (female, vaccinated). Infertility and period irregularities cited in T1, T3, and T6 and concerns due to breast feeding were raised in T1 and T6.

Trust, confusion and uncertainty were themes that contributed toward vaccine hesitancy through many interviews. When asked about key concerns regarding vaccines, Participant 1 (female, not vaccinated) stated “*The information regarding vaccines has been very to and from i.e., this is good for them, this is dangerous. I feel like it is my duty to take care of my son, I feel a bit uneasy with the breastmilk.”*

When asked about the sources of information that the participants have for vaccine related content, many said they do not trust the official sources or are confused. This can be seen in Transcript 3 (female, not vaccinated), which states “*I think it is very confusing. The things they have on official websites, social media talks about it, and you don't know what to trust.”* This is from a Participant who appeared to lose trust in official sources of information due to the content seen on social media. Trust appears to be placed in people the participant knows, as the Participant also states “*I would go to some sort of official website or maybe my uncle. I think the main thing is trusting someone who has more knowledge of this than me.”*

Trust in medical professionals such as General Practitioners (GP) was an influencing factor in the vaccine uptake decision making process. This can be seen in the case of Participant 4 (female, not vaccinated) and Participant 9 (male, vaccinated). Both participants had concerns regarding their allergies presenting side effects if they took the COVID-19 vaccine. Participant 9 called their GP practice and booked an appointment with a nurse who confirmed the vaccine was safe to take, following which the Participant decided to get vaccinated.

Participant 4 did not consult their GP regarding their concerns, and did not get vaccinated, explaining, “*I once went to see the GP because I had some spots on my back, and I was prescribed steroids, and it made the problem worse and I still have the spots now. That makes me think they don't know me and at my age I know my body and what I react to.”* This suggests that previous negative experiences have led to a loss of trust, which may have left the Participant vulnerable to seeking unofficial advice.

### Social Media Impact on Vaccine Hesitancy

Most participants said they received messages on social media regarding COVID-19 vaccines, with the contents being both anti-vaccination and pro-vaccination. The messaging generally happened through WhatsApp, with links to social media content on platforms such as Instagram and YouTube. Some participants said they were impacted by these messages, whilst others said they were not affected. Impacting factors are not always clear; even if a participant is impacted by social media, they may not recognize this. This can be seen in Transcript 11 (male, not vaccinated). When asked about impact of social media on vaccine hesitancy, the answer was “*not at all*” and “*I don't feel peer pressure from either side, to do it or not to do it*” yet the Participant goes on to say “*You see stories on social media about rare blood clotting or rare immune systems. Those stories are very off-putting, because then you see that there is a risk factor to the COVID-19 vaccines.”*

The participants discussed receiving anti-vaccination content concerning topics of extreme side effects such as blood clots, inflammation and negative effects on the immune systems. Participants also cited “conspiracy theories”, including the idea that COVID-19 is not real and microchips were used in vaccines for tracking purposes. While some participants found these theories to be exaggerated, scaremongering or false, others thought there was some truth in them. The official messaging around the COVID-19 vaccinations also elicited various responses from the participants, including feeling suspicious, pressurized to take the vaccine, guilty for not taking the vaccine or finding the volume of advertising of the vaccine frightening. The removal of anti-vaccination content from social media platforms can also add suspicion, as Participant 4 (female, not vaccinated) states “*I go on YouTube, there are some very clever people, some scientists, who are speaking against the vaccinations, but they get banned. That makes you think why are they doing this? Let us make up our mind. I mean everyone is responsible.”*

### Overcoming Vaccine Hesitancy

**Figure 3:**
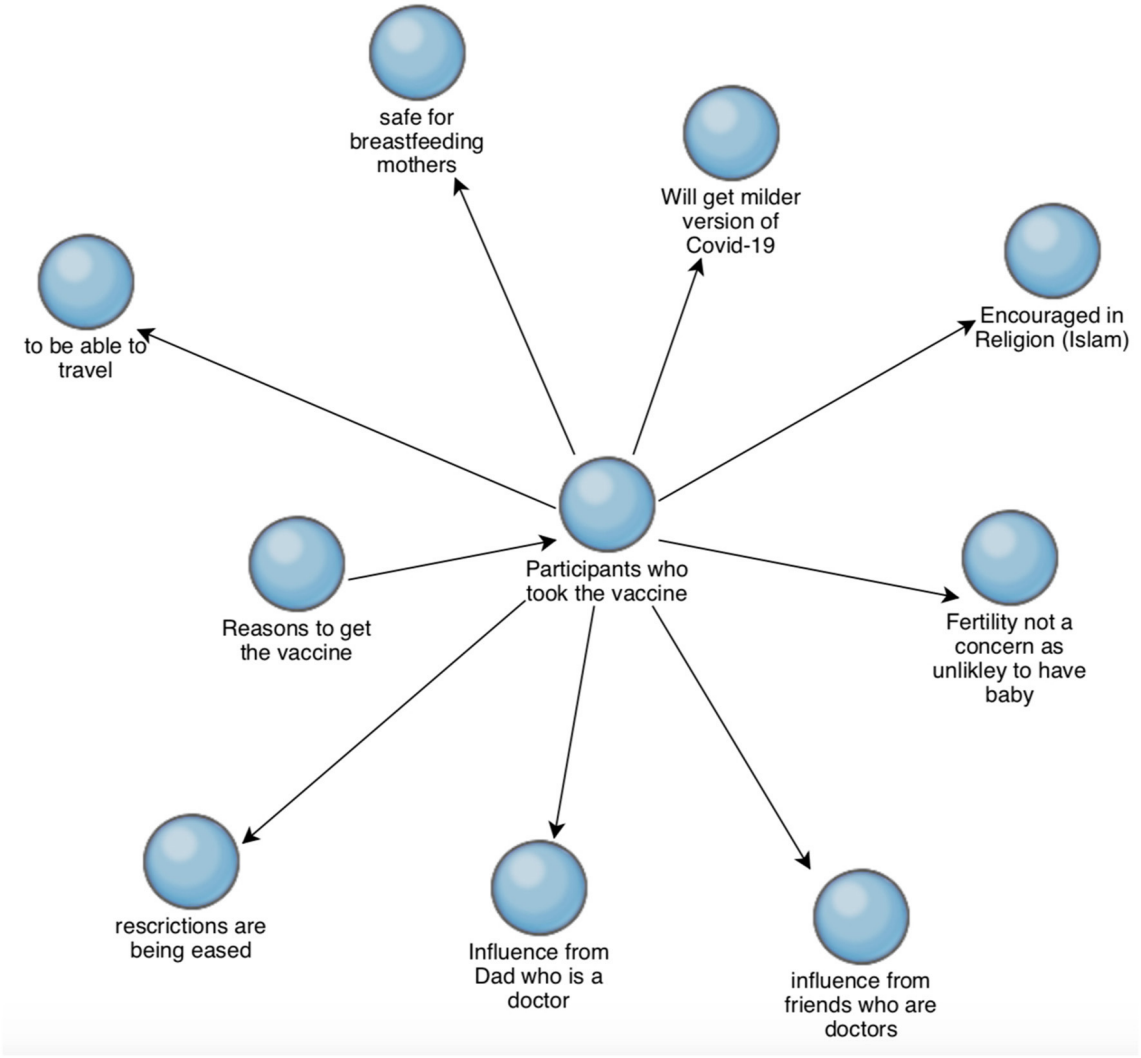
Motivations for potentially receiving the COVID-19 vaccine.

Figure 3 shows reasons that motivated the participants to get vaccinated against COVID-19. Several suggested they know or suspect that they have had COVID-19. For Participants T2 (male, not vaccinated) and T3 (female, not vaccinated), this was one of the reasons to not vaccinate, while for Participant T5 (female, vaccinated), having had COVID-19 has not been a deterrent, as should they get infected again, they expect to get a milder symptoms.

An influencing factor in choosing to get vaccinated against COVID-19 also appears to be having close connections with healthcare professionals. This is most evident in T7 (female, vaccinated), which states; “*My dad is a doctor and he is always talking about vaccines, so I kind of knew that I should get vaccines.”*

**Figure 4:**
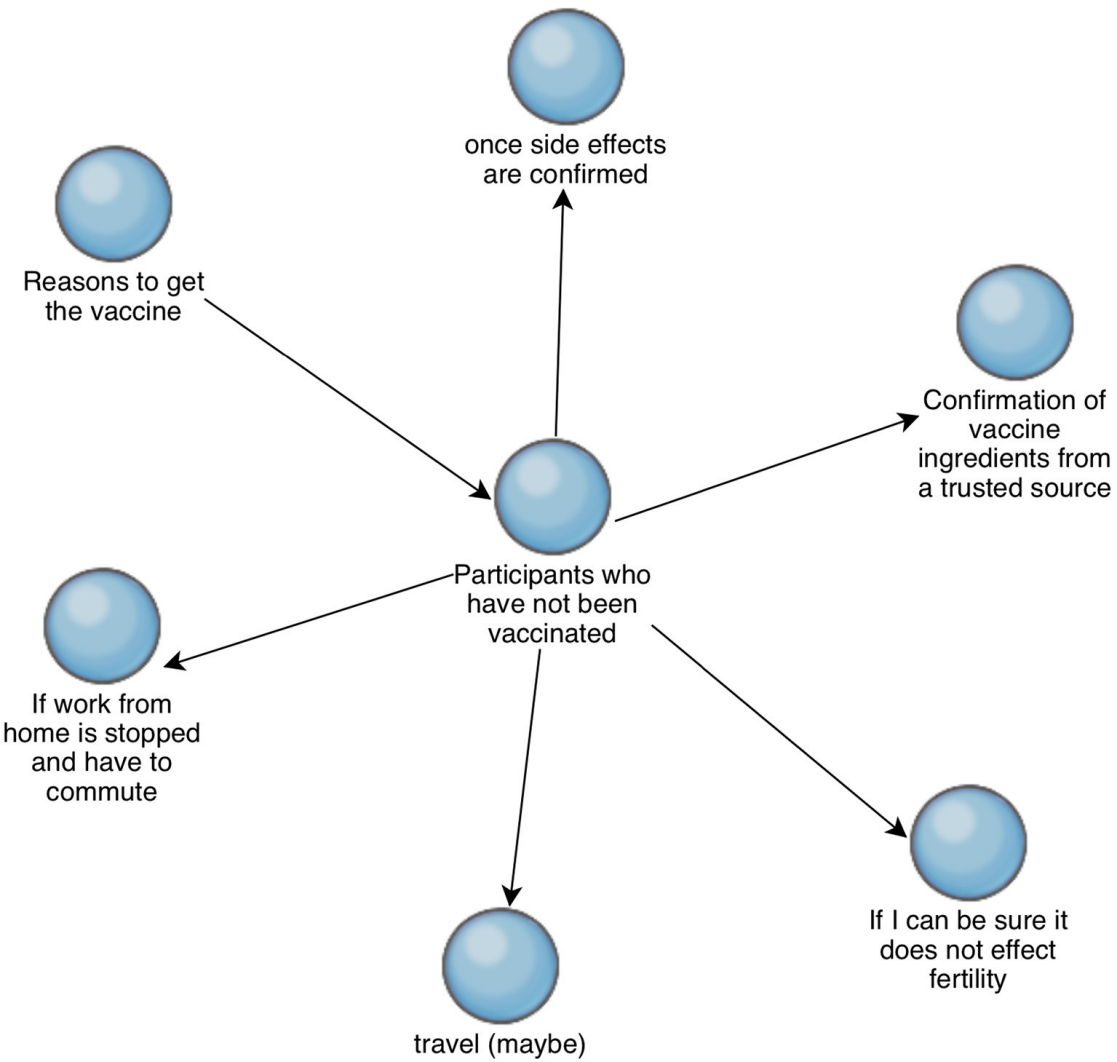
Health Belief Model from Abraham and Sheeran (13).

Figure 4 shows the reasons that participants who have not already received the COVID-19 vaccination may get vaccinated. Of the seven participants who have not been vaccinated, four said that they may take the vaccine if not being vaccinated would hinder travel. The general themes were a need for further clarification or certainty regarding the ingredients in the vaccine, general concerns about side effects, and specific side effects especially with regards to fertility. Participant 1 (female, not vaccinated) said that they may consider taking the vaccine once they have stopped breastfeeding, as side effects to her child would be a major concern.

### Health Belief Model

Considering the categories in the Health Belief Model (13) (Figure 1), the *perceived susceptibility* of COVID-19 amongst the participants who have not been vaccinated appears to be low. The following reasons were provided during interview: wearing appropriate PPE, not using public transport and limiting interactions with large groups. The *perceived severity* of COVID-19 is also low among the participants who have not been vaccinated, as some have recovered from COVID-19 and feel they have developed an understanding of the risks, while others feel their immune system will be protective. Meanwhile the *perceived barriers* to getting a COVID-19 vaccine appear to stem largely from misinformation on social media and lack of access, understanding or trust of authentic sources of information regarding the vaccines. This lack of trust extends to the government and institutions for some participants.

A *perceived benefit* is the ability to travel. The *health motivation* in general appears to be low in both the participants who did get the vaccination against COVID-19 and those who didn't. A reason for this may be that the majority of the participants perceived the risk of extreme side effects from the vaccination to be high. Generally, the participants who chose to get vaccinated considered that the risk from COVID-19 was greater than the risk from highly adverse side effects of the vaccine, while the participants who did not get vaccinated believed the opposite. Examples of a *cue to action* in overcoming vaccine hesitancy for some participants has been friends and families, further reading on websites such as The World Health Organization or simply discussing their concerns with their General Practitioner.

## Discussion

### Reasons for Vaccine Hesitancy

An BMJ study examining why ethnic minority groups are COVID-19 vaccine hesitant based on data from large scale surveys identified that long term side effects and a lack of trust were the primary reasons (17). A Lancet study highlighted similar themes with the addition of risk of deportation when registering for vaccinations and infertility (18). Fertility has been cited elsewhere as a reason for COVID-19 vaccine hesitancy; for example, in a Bradford based study where twenty participants were interviewed, one participant stated infertility as a reason for not taking the vaccine (19). When considering Covid-19 vaccine hesitancy in general in the UK, a nationally representative survey showed the following common reasons for refusal; being against vaccinations, concerns regarding the safety of the vaccines, considering that COVID-19 was harmless and lack of trust generally (20).

In this study, ethnic minorities were specifically investigated, and infertility was found to be a prevalent theme. Including both vaccinated and non-vaccinated participants in the study enabled the understanding of the broad spectrum of infertility related vaccine hesitancy amongst women, which extended to concerns regarding period irregularities and breastfeeding. To clarify the misconception and misinformation around fertility and COVID-19 vaccinations a study was conducted which demonstrated that the Pfizer-BioNTech's vaccination against COVID-19 has no negative effect on a women's fertility (21).

When asked about COVID-19 vaccine type preference, almost all participants said Pfizer was preferred over Astra Zeneca, or Moderna. The participants cited reasons such as the Pfizer vaccine having greater efficacy, concerns regarding risk of blood clots from the Astra Zeneca vaccine and hearing from friends and family that Pfizer has milder side effects. COVID-19 vaccinations and their side effects continue to receive extensive media coverage and this may not have been helped by mixed messages from government bodies. In early March 2021, several European countries paused the use of Oxford-AstraZeneca vaccination against COVID-19, due to some reports of thromboembolic events with fatal outcomes amongst those who had been vaccinated (22). The European Medicines Agency reviewed these events and announced that despite possible link to rare blood clots, the benefits of this vaccination outweigh the risks, following which many countries reinstated their vaccine programs (23), however these concerns persisted amongst participants in our study. Recently, a Spanish cohort study found that rates of thromboembolic events in people who received the Pfizer vaccine were no different to those who received the Astra Zeneca vaccine, though this paper has not yet been peer-reviewed (24).

### Social Media Impact on Vaccine Hesitancy

Social media played a role in contributing toward vaccine hesitancy. For some, particularly those who felt healthy, seeing misinformation of extreme side effects relating to the COVID-19 vaccinations on social media gave the idea that the risk of vaccination is greater than the risk of COVID-19. Misinformation on social media regarding fertility became a reason for delaying or not getting vaccinated. The concerns appear to be further solidified when discussed with friends and families who may have seen similar content on social media, subject to social media echo chambers. Monitoring and removing misinformation from social media platforms has been a solution. However, some participants have shared that this leads them to believe that the government has something to hide.

Better understanding how and who spreads misinformation through social media networks is the key to take action (25), with more research required to analyze and visualize this information in real time (26). As people receive misinformation on social media and are adversely influenced (7), it becomes crucial that they have access to trusted sources of information. Rebuilding of trust must be achieved over time and can start with key points of contact such as local health care providers and community leaders. For instance, many participants did not think they could discuss their concerns regarding vaccinations with their General Practitioner.

This study identified that there is need for further work to be done toward combatting misinformation and conspiracy theories, which can be resolved by social media companies take responsibility for deleting such content from social media, but also by actively responding and then disproving the misinformation.

### Overcoming Vaccine Hesitancy

A factor in choosing to get vaccinated was access and trust of sources of information outside of social media and news outlets. Participants who knew medical professionals or were trusting of their GP or the NHS website appeared more likely to get vaccinated, despite concerns.

Ahead of the launch of the vaccine programme in the UK, studies conducted to predict groups that may be vaccine hesitant identified high vaccine hesitancy amongst ethnic minority communities (11), leading to medical specialists calling for ethnic minorities communities to be considered a priority for vaccination (27) which has not happened (28).

### Implications for Policy and Practice

This study demonstrates that vaccine hesitancy amongst ethnic minorities is a broad spectrum of views, with some participants choosing to get vaccinated against COVID-19 despite their concerns, some waiting for further information or benefit whilst others choosing not to get vaccinated. The results identified many misconceptions regarding COVID-19 vaccinations which need to be addressed or continue to be tackled by governing bodies, academics and public health officials to restore confidence in vaccines, specifically; long term side effects, extreme side effects, vaccine ingredients and fertility.

While this study focuses on vaccine hesitancy in ethnic minority communities, it is important to also consider that linking ethnic minority communities with vaccine hesitancy can result in the incorrect framing of the issue. The link can suggest mistakenly that ethnic minorities are to blame for being vaccine hesitant, rather than focusing on the need for public health systems to be more accessible to all (18).

The reality of the issue is multidimensional with many structural barriers at play. A UK study found that when approaching the police and other local services, twice as many Asian and Black respondents faced discrimination when compared to the White respondents. This study also showed an association with experiences of discrimination and low vaccine uptake (29). Minority groups have also been historically exploited in medical experiments such as the abusive US Tuskegee syphilis study (30). Unfortunately some exploitation continues; for example, a study used experimental drugs on Nigerian children without consent from their parents, a clear ethical violation (31), further giving rise to mistrust.

It is crucial that vaccine hesitancy is not grouped with anti-Vaxxers; this study demonstrates that vaccine hesitancy has a temporality, and can be overcome. The solution does not appear to be as simple as translating vaccine information into multiple languages, but rather involves getting to the core of the issue of mistrust and misinformation, and developing long term, sustainable relationships. Improving vaccine uptake in this way would not only support communities who have been disproportionately impacted by COVID-19 but would also improve immunity in the wider population (18).

The balance of research on vaccine efficacy, safety and hesitancy is primarily focused on western and white populations (32, 33). As a result, public health policy-making and communications may be biased toward these groups. Further research on all aspects of vaccinations for non-white ethnicities—the vast majority of the world's population—is needed to redress these structural imbalances.

### Strengths and Limitations

The interview structure and open ended nature of the questions allowed participants to discuss in the topic in as much detail as they are comfortable with while continuing to develop their answers and thought process. The final question where participants were asked if there is anything further they would like to add provided scope for further points that the participant may have thought of during the interview. A key strength of this study, setting it aside from similar research, was to include participants who had been vaccinated as well as those who were not which allowed for identification of ways vaccine hesitancy may be overcome.

Utilizing established methods of qualitative analysis revealed key themes that can be further explored. Creating a *de novo* framework reduced structural bias of having to fit reasons for vaccine hesitancy into frameworks designed for other groups. While the answers provided by the participants may be representative of some ethnic minority communities, the answers and reasonings for vaccine hesitancy provided in the interviews may not be exclusive to ethnic minority communities. A larger sample size may allow for a greater number of ethnic minorities communities to be included in the study. While the diversity of the sample was wide, it did not fully represent all ethnic minorities, for instance, none of the participants came from a Chinese background. In future studies interpreters may also be allowed for, to include non-English speaking participants.

## Conclusion

This study identified the broad spectrum of views regarding vaccine hesitancy in ethnic minority groups in the UK, and established that vaccine hesitancy may be overcome to varying degrees. Long term side effects as well as side effects in general were the main concerns amongst the twelve participants. Social media plays a role in contributing toward vaccine hesitancy. For some, particularly those who felt healthy, seeing misinformation of extreme side effects relating to the Covid-19 vaccinations on social media resulted in the opinion that the risk of vaccination is greater than the risk of COVID-19. For women, misinformation on social media regarding fertility became a reason for delaying or not getting vaccinated. A factor in choosing to get vaccinated was access and trust of sources of information outside of social media and news outlets. Participants who knew medical professionals or were trusting of their GP or the NHS website appeared more likely to get vaccinated, despite concerns.

Developing and building trust amongst ethnic minorities is often seen as a problem within that community rather than a problem with the public health messaging and approach. Further studies are required to better understand the root causes of the lack of trust government organizations and institutions. The dismissal of vaccination concerns from mainstream discourse and lack of consideration for further transparency, accurate media and social media reporting, and a perceived lack of trusted sources of information appear to increase vaccine hesitancy. Concerted efforts are required to create a truly inclusive vaccination programme. One that does not align with the in-built structural inequalities within our society and healthcare system.

## Data Availability Statement

The original contributions presented in the study are included in the article/supplementary material, further inquiries can be directed to the corresponding authors.

## Ethics Statement

The studies involving human participants were reviewed and approved by UCL Ethics Committee in July 2021, and was given the Project ID 21185/00. The study was also registered with the UCL Data Protection Office, and is registered under reference Z6364106/2021/07/135 social research in line with UCL's Data Protection Policy. The participants provided their written informed consent to participate in this study.

## Author Contributions

MN designed the study and drafted the manuscript. All authors reviewed the study and manuscript and provided critical comments on the draft, and contributed to the interpretation of review findings.

## Funding

This research was partially supported by a research grant funded by the Belmont Foundation, UKRI (Reference number: NE/T013664/1). LL was partially supported by China Scholarship Council (File No. 202008060009).

## Conflict of Interest

The authors declare that the research was conducted in the absence of any commercial or financial relationships that could be construed as a potential conflict of interest.

## Publisher's Note

All claims expressed in this article are solely those of the authors and do not necessarily represent those of their affiliated organizations, or those of the publisher, the editors and the reviewers. Any product that may be evaluated in this article, or claim that may be made by its manufacturer, is not guaranteed or endorsed by the publisher.
